# Willingness to use pre-exposure prophylaxis for HIV prevention among men who have sex with men in Malaysia: Findings from an online survey

**DOI:** 10.1371/journal.pone.0182838

**Published:** 2017-09-13

**Authors:** Sin How Lim, Gitau Mburu, Adam Bourne, Joselyn Pang, Jeffrey A. Wickersham, Clayton Koh Thuan Wei, Ilias Adam Yee, Bangyuan Wang, Matteo Cassolato, Iskandar Azwa

**Affiliations:** 1 Center of Excellence for Research in AIDS (CERiA), Faculty of Medicine, University of Malaya, Kuala Lumpur, Malaysia; 2 Division of Health Research, University of Lancaster, United Kingdom; 3 Australian Research Centre in Sex, Health & Society, La Trobe University, Melbourne, Australia; 4 Sigma Research, Department of Social & Environmental Health Research, London School of Hygiene & Tropical Medicine, London, United Kingdom; 5 International Programs, Australian Federation of AIDS Organizations, Bangkok, Thailand; 6 Yale School of Medicine, Department of Internal Medicine, Section of Infectious Diseases, AIDS Program, New Haven, CT, United States of America; 7 Malaysian AIDS Council, Kuala Lumpur, Malaysia; 8 Infectious Diseases Unit, Faculty of Medicine, University of Malaya, Kuala Lumpur, Malaysia; University of Toronto, CANADA

## Abstract

**Objective:**

We examined willingness to use pre-exposure prophylaxis (PrEP) for HIV prevention among men who have sex with men (MSM) in Malaysia.

**Methods:**

An online survey of 990 MSM was conducted between March and April 2016. Eligibility criteria included being biological male, Malaysian citizen, 18 years of age or above, identifying as MSM, and being HIV negative or unknown status. Participants’ demographics, sexual and drug use behaviors, attitudes towards PrEP, and preferences regarding future access to PrEP were collected. Bivariate analysis and logistic regression were performed to determine factors associated with willingness to use PrEP.

**Results:**

Fewer than half of participants (44%) knew about PrEP before completing the survey. Overall, 39% of the sample were willing to take PrEP. Multivariate logistic regression indicated that Malay men (AOR: 1.73, 95% CI:1.12, 2.70), having 2 or more male anal sex partners in the past 6 months (AOR: 1.98, 95% CI: 1.29, 3.05), previous knowledge of PrEP (AOR: 1.40, 95%CI: 1.06, 1.86), lack of confidence in practising safer sex (AOR: 1.36, 95% CI: 1.02, 1.81), and having ever paid for sex with a male partner (AOR: 1.39, 95% CI: 1.01, 1.91) were independently associated with greater willingness to use PrEP, while men who identified as heterosexual were less willing to use PrEP (AOR, 0.36, 95% CI: 0.13, 0.97). Majority of participants preferred to access PrEP at affordable cost below 100 Malaysian Ringgit (USD25) per month from community based organisations followed by private or government hospitals.

**Conclusions:**

Overall, MSM in Malaysia reported a relatively low level of willingness to use PrEP, although willingness was higher among those previously aware of PrEP. There is a need to provide PrEP at affordable cost, increase demand and awareness of PrEP, and to provide access to this preventative medication via diverse, integrated and tailored sexual health services.

## Introduction

The safety and efficacy of oral antiretroviral drugs in reducing the risk of HIV infection has been demonstrated in 15 randomized control trials and 3 observational studies across different populations globally [1]. The World Health Organization (WHO) initially recommended the use of oral Pre-Exposure Prophylaxis (PrEP) to reduce HIV acquisition by HIV-negative partners within serodiscordant heterosexual couples in 2012 [2]. In 2015, WHO expanded this recommendation to include oral PrEP as part of comprehensive HIV prevention for key populations, including men who have sex with men [3]. Currently, the United States, Canada, France, Kenya, Peru, and South Africa have approved PrEP. Other countries, particularly in the Asia Pacific region, have embarked on several PrEP implementation projects [4].

The success of PrEP implementation will depend on its widespread acceptability and access among those who need it. In recent years, research on the acceptability of PrEP has increased significantly. Two systematic reviews identified more than 30 quantitative studies on acceptability or willingness to use PrEP [5, 6]. A recent meta-analysis estimated the acceptability of using PrEP among MSM to be 57.8% globally [7]. Outside of Asia, research on acceptability of PrEP among MSM has been conducted mostly in the United States of America [8–22], followed by United Kingdom [23–26], Australia [27–29], Brazil [30], Canada [31–33], France [34, 35], Kenya [36], Netherlands[37], Peru [38], Portugal [39], Switzerland[40], and Spain [41]. Other studies have assessed acceptability of PrEP using surveys that included MSM from multiple countries [42, 43]. In Asia, acceptability research among MSM has been conducted in China [44–47], Taiwan [48], Thailand [49–51], Myanmar [52], and Vietnam [53]. A review of these studies identified differing levels of willingness to use PrEP by MSM, and generally low levels of awareness of PrEP among Asian MSM [54].

In Malaysia, although PrEP has been included as part of the Ministry of Health ‘National Strategic Plan to end AIDS by 2030’, antiretroviral drugs are currently not licensed for HIV prevention [55]. A national survey conducted by the Ministry of Health found that between 2012 and 2014, HIV prevalence among MSM had increased from 7.1% to 8.8% [55]. The rising prevalence among MSM can be explained by both high levels of risk behaviors [56, 57], and a low uptake of HIV testing [57] in this population. Therefore, many MSM may not be aware of their infection and are not initiated on antiretroviral treatment. In addition, the Malaysian law, specifically the Section 377 of its Penal Code, criminalizes same sex intercourse: introduction of the penis into the anus or mouth of the other person is classified as carnal intercourse against the order of nature, which is punishable with imprisonment of up to twenty years, and is liable to whipping [58]. This harsh law is a documented impediment to HIV prevention and outreach activities for MSM in Malaysia [58].

Although numerous acceptability studies have been conducted among MSM, research in this area is still in its infancy in Asian countries, including Malaysia. In addition, a consistent finding in existing PrEP research is that demographic as well as contextual socio-economic, cultural, and structural factors may influence the acceptability and potential uptake of PrEP among MSM in Asia and globally [7, 54], which limits universal generalisability of findings from existing studies. In the Malaysian context, PrEP can be a useful intervention for MSM who experience problems using condoms, or struggle to use them consistently, as well as those who engage in risky behaviors such as multiple casual sex partners [57] and recreational drug use in the context of sex [59]. Despite the inclusion in 2015 of PrEP in the National Strategic Plan to End AIDS by 2030, PrEP implementation projects are not due to start until late 2017, partly due to uncertainties regarding potential demand, cost implications, and implementation modalities. Given the rising HIV prevalence among Malaysian MSM, it is important to understand how PrEP can be implemented as part of targeted combination prevention services for this population. Therefore, the aim of this study was to assess willingness to use PrEP among MSM, as well as factors associated with such willingness. We also examined the features of PrEP services desired by MSM in Malaysia.

## Methods

### Study design

As part of a two-phase study, an online MSM PrEP survey was conducted between 15 March and 16 April 2016. The second phase of the study involved focus group discussions among a sub-sample of survey participants [60]. This paper presents data only from the first phase of the study.

### Study procedures

Between 15 March and 16 April 2016, 990 MSM completed the Online MSM PrEP Survey. Study participants comprised a convenience sample of MSM recruited via advertisements on mobile apps that target MSM communities, including Grindr, as well as MSM who were connected to local HIV/AIDS community-based organizations. The study was advertised through a banner on Grindr, a popular social networking app for MSM in Malaysia. Apart from recruitment through gay mobile apps, outreach workers and staff members of community based organization (CBO) additionally promoted the study to MSM in their social networks to encourage participation. The anonymous self-administered online questionnaire took approximately 15–20 minutes to complete.

**Fig 1:**
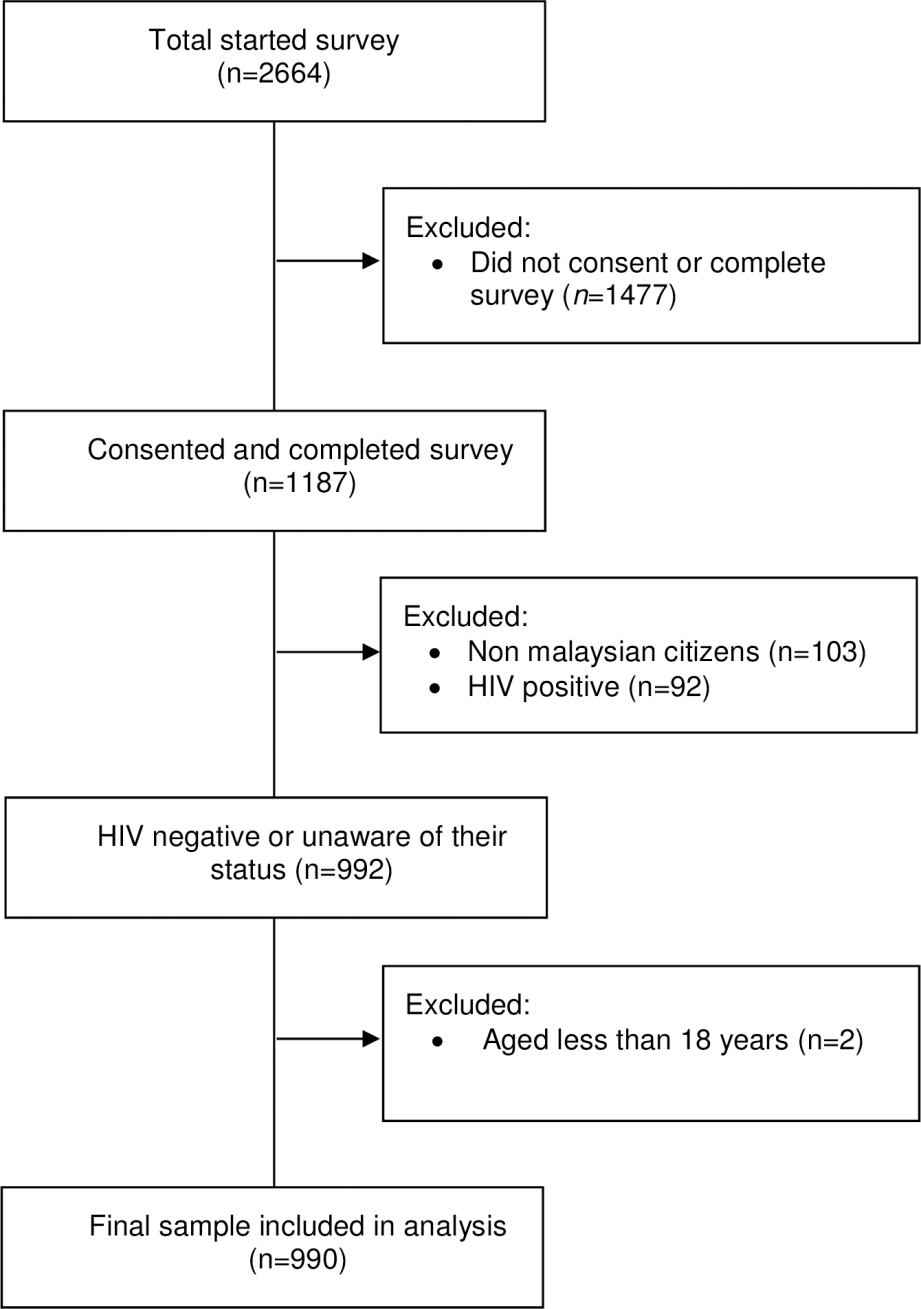
Participant selection flow diagram.

Participants were eligible if they self-reported to be male, a Malaysian citizen, 18 years old or above, negative or unknown HIV status, and identified as men who have sex with other men. Participants who did not report sex with other men or self-identify as MSM were excluded from the survey. The survey was programmed and delivered via Survey Monkey [61]. During the one-month recruitment period, 2,664 participants entered the survey and 1,187 (44.6%) consented and completed the questionnaire. Of 1,187 men, 1,084 identified themselves as Malaysian citizens. The sample was further limited to 992 men who reported to be HIV-negative or of unknown status. Of the 992 men, two were excluded because they were under the age of 18. The final sample consisted of 990 MSM who fulfilled the eligibility criteria. The participant selection is shown in Fig 1.

### Measures

The Online MSM PrEP survey included questions on demographic characteristics, HIV and sexually transmitted infection (STI) testing and diagnosis, and sexual and drug use behaviors. The following definitions were used for analyses: previous knowledge of PrEP was defined as having known that PrEP was an effective HIV prevention intervention prior to survey participation; ever received HIV testing was defined as having been HIV tested at least once in their lifetime; had a serodiscordant sexual partner was defined as having engaged in sexual activity with a man you knew to have diagnosed HIV in the last 6 months; STI diagnosis was defined as having been diagnosed with any STI other than HIV in the last 12 months; inconsistent condom use was defined as having any insertive or receptive anal intercourse without a condom in the last 6 months, and as a result, MSM who did not have any anal sex male partners were included under the converse category of consistent condom users. Participants were asked if they had ever taken any recreational drugs (cannabis, cocaine, “foxy” [5-methoxy-N,N-diisopropyltryptamine (5-MeO-DiPT), GHB [γ-hydroxybutyrate], heroin, crystal meth or “ice”, LSD [Lysergic acid diethylamide], “magic mushroom”, poppers, or Viagra/Cialis), followed by a separate question that assessed the frequency of taking recreational drugs before or during sex (‘chem sex’) in the past 6 months. The frequency of ‘chem sex’ was then dichotomized for the purpose of the analysis.

To assess participants attitudes regarding their perceived likelihood of contracting HIV, participants were asked to rate their agreement to the following statement: “it is likely that I will contract HIV within the next 12 months”. Likewise, to assess participants’ confidence in practising safer sex, respondents were asked to rate their agreement to the statement: “the sex I have is always safe as I want it to be”. Additionally, to evaluate participants’ communication with healthcare providers, they were required to rate their agreement to the statement: “I feel comfortable telling my doctor about my sexual behavior”. These attitudinal questions were asked on a 5-point Likert-type scale (1 = strongly agree to 5 = strongly disagree), but their measures were dichotomized for analytic purposes to either “strongly agree/agree or neutral/disagree/strongly disagree”.

Additionally, participants were asked if they had ever heard of or taken PrEP and/or PEP. Previous PEP utilization was defined as having taken PEP in the last 12 months (“yes/no”), and this question was preceded by a definition of PEP. To provide a common understanding of PrEP as well as a clear distinction between PrEP and PEP, a description of PrEP was also provided: “*PrEP is a daily medication that people who do not have HIV take to prevent getting infected with HIV*. *PrEP is taken before someone is exposed to HIV*.*”* It was emphasized that PrEP is most beneficial to individuals at higher risk of contracting HIV and for those who have difficulty in using traditional prevention methods, such as consistent condom use. Participants were also reminded of potential side effects and the need for regular follow up and adherence to medication for PrEP to be successful.

The primary outcome of the study was “willingness to use PrEP” using a shorter version of the 7-item scale developed by Holt et al. [27]. A 5-item scale (α = 0.92) was constructed using the following items: 1) “I would need to take PrEP”, 2) “I would take PrEP even if it wasn’t 100% effective”, 3) “I am going to take PrEP as soon as it becomes available”, and 4) “I would take pills before or after sex if it would prevent me getting HIV”, and 5) “I would take a pill every day if it would prevent me from getting HIV”. Each item was scored from 1 = very unwilling to use to 5 = most willing to use. Participants whose mean scored ≥ 4 on the 5-item scale were categorized as willing to use PrEP.

The secondary outcomes of the study include variables related to the access and delivery of PrEP services. For example, the amount of money participants were willing to spend on PrEP and the venue they would prefer to access it. Moreover, participants were asked about their preferred dosing strategy (daily versus event-based), and their understanding of the difference between PrEP and PEP.

### Statistical analysis

Bivariate analyses were conducted to compare differences in demographics, HIV testing, STI diagnosis in the past 12 months, recreational drug use before or during sex (“chem sex”), and other HIV-related behaviors between MSM who were willing and those who unwilling to use PrEP using chi-square test or t-test. Factors that were significantly associated with willingness to use PrEP in the bivariate analysis (*p*<0.1) were purposely selected and further explored using a multivariable logistic regression model [62, 63]. Multicollinearity of the predictor variables were assessed and assumption of multicollinearity was not violated. All statistical analyses were conducted using SPSS version 23.0 (SPSS Inc., Chicago, IL).

### Ethical considerations

Each participant completed an online informed consent form by acknowledging that they understood the purpose, eligibility criteria, risks and benefits of the study. The Medical Ethics Committee of University of Malaya approved the study (MECID No: 20161–2010).

## Results

### Participants’ characteristics

Almost half of the participants were ethnic Chinese and most completed the survey in English. The mean age was 30.60 (range 18–68) years. The majority of participants were single, identified themselves as gay, highly educated and working full time (see Table 1). Participants came from all states of Malaysia including East Malaysia. The majority of participants reported having confidence in maintaining safer sex and most were not comfortable in talking about sexual behaviors with their health providers. About one third of participants had never tested for HIV. More than 85% of participants did not know their STI status in the past 12 months (see Table 2).

**Table 1:** Demographic characteristics and willingness to use PrEP.

	Total (N = 990)	Unwilling to use PrEP (n = 603)	Willing to use PrEP (n = 387)	χ^2^ or *t*-test	df	*p*
Age, mean (SD)	30.6 (7.5)	30.7 (7.8)	30.5 (7.1)	0.35		0.75
Race						
Malay	383 (38.7%)	197 (51.4%)	186 (48.6%)	23.90	3	<0.001
Chinese	486 (49.1%)	327 (67.3%)	159 (32.7%)			
Indian	61 (6.2)	41 (67.2%)	20 (32.8%)			
Other/mixed	60 (6.1%)	38 (63.3%)	22 (36.7%)			
Residence						
Greater Kuala Lumpur	679 (68.6%)	419 (61.7%)	260 (38.3%)	1.38	2	0.502
Other states of West Malaysia	273 (27.6%)	159 (58.2%)	114 (41.8%)			
East Malaysia	38 (3.8%)	25 (65.8%)	13 (34.2%)			
Language of survey						
English	672 (67.9%)	424 (63.1%)	248 (36.9%)	5.01	2	0.082
Malay	237 (23.9%)	130 (54.9%)	107 (45.1%)			
Chinese	81 (8.2%)	49 (60.5%)	32 (39.5%)			
Sexual identity						
PLU/Homosexual/Gay	796 (80.4%)	481 (60.4%)	315 (39.6%)	6.63	2	0.036
Bisexual	164 (16.6%)	97 (59.1%)	67 (40.9%)			
Straight/Heterosexual	30 (3%)	25 (83.3%)	5 (16.7%)			
Education						
Secondary or lower	127 (12.8%)	80 (63.0%)	47 (37.0%)	1.81	2	0.405
College/university/professional degree	649 (65.6%)	401 (61.8%)	248 (38.2%)			
Post graduate	214 (21.6)	122 (57.0%)	92 (43.0%)			
Employment status						
Student	148 (14.9%)	85 (57.4%)	63 (42.6%)	2.31	2	0.315
Full time	671 (67.8%)	406 (60.5%)	265 (39.5%)			
Part time/self- employed/unemployed/retired	171 (17.3%)	112 (65.5%)	59 (34.5%)			
Monthly Income						
< RM2000 (USD500)	268 (27.1%)	174 (64.9%)	94 (35.1%)	2.49	1	0.115
≥RM2000 (USD500)	722 (72.9%)	429 (59.4%)	293 (40.6%)			
Relationship status						
Single	595 (60.1%)	356 (59.8%)	239 (40.2%)	0.91		0.634
In a relationship	324 (32.7%)	201 (62.0%)	123 (38.0%)			

PLU = people like us, a code word for gay or homosexual used by men who have sex with men in Malaysia, df = degrees of freedom, RM = Malaysian Ringgit, SD = standard deviation, USD = United States dollar, χ^2^ = chi-square, *p* = p-value.

**Table 2:** HIV-related behavioral characteristics and willingness to use PrEP.

	Total (N = 990)	Unwilling to use PrEP (n = 603)	Willing to use PrEP (n = 387)	χ^2^	df	*p*
Previously heard of PrEP						
Yes	432 (43.6%)	248 (57.4%)	184 (42.6%)	3.95	1	0.047
No	558 (56.4%)	355 (63.6%)	203 (36.4%)			
Ever received HIV Testing						
Yes	661 (66.8%)	404 (61.1%)	257 (38.9%)	0.04	1	0.847
No	329 (33.2%)	199 (60.5%)	130 (39.5%)			
Had serodiscordant sexual partner(s)*						
Yes	41 (4.1%)	25 (61.0%)	16 (39.0%)	0.0	1	0.993
No	949 (95.9%)	578 (60.9%)	371 (39.1%)			
Number of male anal sex partner*						
0	174 (17.6%)	127 (73.0%)	47 (27.0%)	24.9	2	<0.001
1	180 (18.2%)	125 (69.4%)	55 (30.6%)			
2 or more	636 (64.2%)	351 (55.2%)	285 (44.8%)			
Any inconsistent condom use*						
Yes	501 (50.6%)	289 (57.7%)	212 (42.3%)	4.43	1	0.035
No	489 (49.4%)	314 (64.2%)	175 (35.8%)			
Diagnosed with STI in the past 12 months						
Yes	141 (14.2%)	80 (56.7%)	61 (43.3%)	1.20	1	0.273
No/Don’t know	849 (85.8%)	523 (61.6%)	326 (38.4%)			
Perceived likelihood of contracting HIV in the next 12 months						
Very likely/Likely	147 (14.8%)	80 (54.4%)	67 (45.6%)	3.05	1	0.081
Very unlikely/Unlikely/Neutral	843 (85.2%)	523 (62.0%)	320 (38.0%)			
Confidence in practising safer sex						
Yes	610 (61.6%)	392 (64.3%)	218 (35.7%)	7.51	1	0.006
No	380 (38.4%)	211 (55.5%)	169 (44.5%)			
Comfortable in communication with healthcare provider about sexual behaviors						
Yes	402 (40.6%)	249 (61.9%)	153 (38.1%)	0.30	1	0.582
No	588 (59.4%)	354 (60.2%)	234 (39.8%)			
Received PEP in the past 12 months						
Yes	18 (1.81%)	9 (50%)	9 (50%)	0.92	1	0.338
No	972 (98.2%)	594 (61.1%)	378 (38.9%)			
Chem sex*						
Yes	170 (17.2%)	94 (55.3%)	76 (44.7%)	2.72	1	0.099
No	820 (82.8%)	509 (62.1%)	311 (37.9%)			
Ever sold sex to a male partner						
Yes	118 (11.9%)	71 (60.2%)	47 (39.8%)	0.03	1	0.861
No	872 (88.1%)	532 (61%)	340 (39%)			
Ever paid for sex with a male partner						
Yes	237 (23.9%)	124 (52.3%)	113 (47.7%)	9.65	1	0.002
No	753 (76.1%)	479 (63.6%)	274 (36.4%)			

*In the past 6 months, df = degrees of freedom, HIV = human immunodeficiency virus, STI = sexually transmitted diseases, PrEP = pre-exposure prophylaxis, PEP = post-exposure prophylaxis, χ^2^ = chi-square, *p* = p-value

In terms of sexual behaviors, 17.6% did not have anal sex with a male sex partner in the past 6 months, and 4.1% reported that they had had sex with serodiscordant male partners. Close to two thirds had 2 or more sexual partners in the last 6 months and half engaged in inconsistent condom use. About 17% had taken recreational drugs before or during sex (colloquially referred to as “chem sex”) and almost a quarter had ever paid for sex with a male partner.

### Awareness of PrEP and variables related to PrEP and PEP

Regarding awareness of PrEP, 44% had heard of PrEP prior to the survey and the main source of such information was the Internet (see Table 3). Ten participants reported to have ever used PrEP and four were currently using it at the time of completing the survey. Of the ten participants who had used PrEP, nine rated their experience as ‘very good’ to ‘satisfactory’ (data not shown). A small minority (1.8%) reported to have use PEP in the past 12 months (Table 2). About one third of participants indicated willingness to pay out-of-pocket for PrEP. Of these, 88% were willing to pay below Malaysian Ringgit (RM) 200 (USD50) per month for the medication. The majority of participants believed that the government should cover the cost of PrEP. The three preferred facilities to access PrEP, in rank order, were community-based organizations, general practitioners (private physicians), and government clinic or hospitals. Close to half of the participants reported that they would only take PrEP as contingency for high-risk sex.

**Table 3:** Knowledge on PrEP and preference of access to PrEP (n = 990).

First learnt about PrEP (among those who have heard of PrEP, n = 432)	
Newspapers and magazines	16 (3.7%)
Internet	307 (71.1%)
Professional journals	22 (5.1%)
Friends	49 (11.3%)
Doctors	15 (3.5%)
Other	23 (5.3%)
Ever Used PrEP	
Yes	10 (1.0%)
No	422 (42.6%)
Willing to pay for PrEP	
Yes	352 (35.6%)
No	165 (16.7%)
Don’t know/not sure	473 (47.8%)
Amount willing to spend per month for PrEP (among those who were willing to pay, n = 352)	
Less than RM 100 (USD25)	187 (53.1)
Between RM 100 and RM 400 (USD25 and USD100)	155 (44.0)
More than RM 400 (USD100)	10 (2.8)
Institution expected to cover the cost of PrEP (multiple choice)	
Government	826 (83.4%)
Private health insurance	414 (41.8%)
Preferred location for accessing PrEP (among those who would likely use PrEP if PrEP was made available in Malaysia, n = 797)	
Private clinic	205 (25.7%)
Private hospital	48 (6.0%)
Government clinic/ Hospital	177 (22.2%)
Community-based health clinics	87 (10.9%)
Community-based organisation	210 (26.3%)
Other	70 (8.8%)
Opinion on PrEP regimen	
“I will continue taking PrEP every day”	298 (30.1%)
“I will only take PrEP before and after high-risk sex”	474 (47.9%)
“I am unsure about when and how to take PrEP”	218 (22.0%)
Understand the difference between PrEP and PEP	
Yes	527 (53.2%)
No	215 (21.7%)
Unsure	248 (25.1%)

Abbreviations: PrEP = pre-exposure prophylaxis, RM = Malaysian Ringgit, USD = United States dollars.

### Willingness to use PrEP

Overall, 39% (n = 387) of men had a mean score of ≥4 in the 5-item ‘willingness to use PrEP’ scale. The means of individual scale items ranged from 3.33 to 3.63 and the mean of the overall scale was 3.46 (standard deviation = 1.03). As each item was scored from 1 = “very unwilling to use” to 5 = “very willing to use”, grand mean of 3.46 indicated that on average, MSM in the present study were slightly above neutral in their endorsement of willingness to use PrEP.

In the bivariate analysis, ethnicity, gay sexual identity, multiple male sexual partners, having any inconsistent condom use in the past 6 months, lack of confidence in practising safer sex, having heard of PrEP, and ever paid for sex with a male partner were significantly associated with willingness to use PrEP. Perceived likelihood of contracting HIV and ‘chem sex’ were marginally associated with willingness to use PrEP.

In the multiple logistic regression model, Malay ethnicity, gay sexual identity, having 2 or more male sex partners in the past 6 months, having heard of PrEP, having a lack of confidence in practising safer sex, and having ever paid for sex with a male partner were independently associated with willingness to use PrEP (Table 4).

**Table 4:** Simple logistic and multivariable logistic regression.

	OR (95% CI)	*p*	AOR^1^	*p*
Malay ethnicity (vs. others)	1.91 (1.47, 2.48)	<0.001	1.73 (1.12, 2.70)	0.015
Chinese ethnicity (vs. others)	0.59 (0.46, 0.76)	<0.001	0.83 (0.54, 1.29)	0.407
Sexual identity				
Gay	Ref		ref	
Bisexual	1.06 (0.75, 1.49)	0.760	1.06 (0.73, 1.52)	0.763
Straight/Heterosexual	0.31 (0.12, 0.81)	0.017	0.36 (0.13, 0.97)	0.043
Number of male anal sex partner*				
0	Ref		ref	
1	1.19 (0.75,1.89)	0.462	1.18 (0.71, 1.97)	0.527
≥ 2	2.19 (1.52, 3.17)	<0.001	1.98 (1.29, 3.05)	0.002
Any inconsistent condom use*				
Yes	1.32 (1.02, 1.70)	0.035	0.93 (0.68, 1.27)	0.641
No	ref		ref	
Heard of PrEP				
Yes	1.30 (1.00. 1.68)	0.047	1.40 (1.06, 1.86)	0.018
No	ref		ref	
Perceived likelihood of contracting HIV in the next 12 months				
Yes	ref		ref	
No	0.73 (0.51, 1.04)	0.081	0.80 (0.55, 1.16)	0.239
Confidence in practising safer sex				
Yes	ref		ref	
No	1.44 (1.11, 1.87)	0.006	1.36 (1.02, 1.81)	0.036
Ever paid for sex with a male partner				
Yes	1.59 (1.19, 2.14)	0.002	1.39 (1.01, 1.91)	0.043
No	ref		Ref	
Chem sex*				
Yes	1.32 (0.95, 1.85)	0.100	1.06 (0.74, 1.53)	0.746
No	ref		ref	

^1^Adjusted for age and education, the goodness of fit of the multivariable model was evaluated by Homer and Lemeshow Test, χ^2^ = 3.372, df = 8, *p* = 0.909.

Abbreviations: OR = Odds Ratio, *p* = p value, PrEP = Pre-Exposure Prophylaxis, USD = United States Dollars, vs = versus.

*in the past 6 months

## Discussion

This is the first study to assess willingness to use PrEP among a key population in Malaysia. The present study found that willingness to use it was related to multiple factors, including demographic characteristics, sexual identity, HIV risk behaviors and prior awareness of PrEP. Our study found that Malay respondents were more willing to use PrEP compared to MSM of other ethnic backgrounds. These ethnic differences in willingness to use PrEP could not be explained by socio-economic factors such as education level, age or income. Other studies have found ethnic differences in health profiles of Malaysians [64, 65], which have been attributed to cultural, health-seeking and lifestyle differences [64–66]. However, the extent to which these factors contribute to our observed differences in willingness to use PrEP is not clear. Other social, ethno-cultural and religious factors should be explored in future studies to understand factors contributing to differential willingness to use PrEP among different ethnic MSM populations.

The finding that MSM who identified as homosexual or gay (versus bisexual/heterosexual) were more willing to use PrEP was similar to a study of MSM in Vietnam [53]. It is possible that MSM who were more ‘out’ or comfortable about their sexual identity were more likely to access HIV prevention services. In other contexts, ‘outness’ has been associated with reduction in sexual risks [67, 68], possibly because MSM who conceal their sexual orientation may feel greater pressure to have their sexual needs met under riskier conditions [68], or because MSM who identify as gay men may be more likely to access social support and educational interventions tailored to this group, which bisexual men may not be reached with [68]. However, the positive relationship between outness and uptake of health services is dependent on the context within which sexual minorities live [68]. For instance, ‘outness’ and associated homophobia was found to reduce willingness to access PrEP and other services in a multi-country study [43], and another study from Kenya found that bisexual participants were more willing to use PrEP compared to MSM who self-identified as homosexual or gay [36]. In contrast, two studies from China did not find an independent association between sexual orientation and willingness to use [44, 47]. Hence, identifying potential high-risk users may require a wide variety of ways to reach both homosexual and bisexually active men.

The present study found that risk behaviors such as having more male sexual partners and paying for sex were independently associated with willingness to use PrEP, echoing findings from China [47]. Taken altogether, our data suggest that PrEP is preferred and should be offered to MSM who are at higher risk of acquiring HIV, in keeping with WHO recommendations [3]. Indeed, PrEP itself can serve as an entry point to HIV testing, STI screening, and other sexual health services, given that MSM who were not aware of their STI status were found to have high levels of willingness to use it.

The level of willingness to use PrEP reported here is similar to recent studies in Thailand [51] and Myanmar [52] which reported level of willingness to use PrEP of 36% and 39%, respectively. Notably, Stoové et al. used the original scale from Holt et al. [27] to measure willingness to use PrEP. An online MSM study from Taiwan used an even shorter version of the scale (4-item) and found that 56% of survey respondents were willing to use PrEP [48]. It must be noted that the variability of level of willingness to use PrEP among MSM in various countries may be due to the variability in measurement [54]. Most of previous studies measured willingness to use PrEP based on one single question and have generally yielded higher percentage of acceptability [54]. Interestingly, the level of willingness to use PrEP of the present study is higher than Holt et al.’s study conducted in 2011 (28.2%) which is among the lowest reported in the studies of MSM in Western countries.

Our results have several implications for eventual demonstration and subsequent widespread implementation of PrEP. First, levels of awareness and knowledge about PrEP remain low among MSM. Half of the participants in our study reported that they did not know or were unsure of the difference between PrEP and PEP even after detailed descriptions of PrEP and PEP were provided. Framing PrEP prevention messages appropriately based on accurate information with support of community partners will be crucial to raise awareness and interest among MSM. Evidence of PrEP efficacy and safety should be widely disseminated to allay concerns held by MSM about potential side effects.

Second, we found that off-label use of PrEP among MSM, although rare, is occurring in Malaysia. Local guidelines and policies are needed to determine eligibility criteria for PrEP and to ensure that antiretroviral drugs are licensed for prevention and can be prescribed safely. Furthermore, it may be useful to position PrEP as a prevention strategy for all populations at risk for HIV infection, including serodiscordant heterosexual couples, in order to avoid further stigmatization of MSM and other key populations.

In our study, the majority of respondents were not willing to pay for PrEP out of pocket. Currently, in Malaysia, available PrEP formulations of emtricitabine and tenofovir disoproxil fumarate (e.g. Tenvir-EM, Cipla) costs as high as RM 800 (USD200) per month at private clinics and about RM 160 (USD40) per month at the government hospitals, although these medications are not currently licensed for HIV prevention. Similar to reports from other Asian settings [48, 52, 69], the cost of medications and routine testing may remain a significant barrier to uptake of PrEP in Malaysia. In terms of service provision, the Ministry of Health and HIV/AIDS CBOs will have to consider how PrEP can be integrated with existing HIV prevention services, such as HIV testing, education, and outreach. This is particularly important because most of the participants in our study reported low level of access to HIV and STI services, as evidenced by the small proportion who were aware of their HIV and STI status.

Garnering support and commitment from HIV care providers will be important in the delivery of PrEP. In this study, about 60% of participants did not feel comfortable talking to their physicians about their sexual behaviors, and only a small minority of men had learnt about PrEP from their physicians. A previous study documented high levels of stigma toward MSM among future healthcare providers in Malaysia [70]. In a global survey of MSM, homophobia, stigma, and service provider stigma were significantly associated with reduced access to services [43]. Prevention strategies such as PrEP may further fuel the perceptions of MSM engaging in condomless sex as selfish, irresponsible, and reckless. A qualitative study from India showed that a major barrier to potential use PrEP among MSM was fear of being stigmatized and labelled as promiscuous by their peers [69, 71]. Therefore, HIV care providers need training not only to increase their knowledge and competency regarding PrEP, but also to dispel negative stigma against potential PrEP users. Effective provider-client communication around gay men’s sexual health will be critical in ensuring that PrEP is provided via a non-judgemental approach. HIV providers are not the only important constituency. Training and competency development in relation to PrEP would also be valuable to primary care providers and other sexual health clinic staff. As highlighted in a recent discussion paper on the rollout of PrEP in the Asia Pacific region [4], the epidemic-limiting potential of this new prevention intervention will only be realized with significant health system investment and with the participation of MSM organizations in the design and delivery of PrEP related policies and programmes.

Several limitations need to be acknowledged. Study participants comprised a convenience sample of MSM who used gay social media or were connected to local HIV/AIDS CBOs and are therefore not representative of all MSM in Malaysia. The study also excluded transgender participants, although we recommend targeted research among this distinct key population to adequately address their specific perspective and needs. In addition, online participation may have excluded MSM without access to internet sites on which the study was advertised.

Our sample differed from other MSM nationally in regard to ethnic profile as well as several HIV risk characteristics. In terms of ethnicity, Chinese MSM were over-represented in the sample even though ethnic Malay is the majority ethnic group in Malaysia. In terms of HIV risk characteristics, 56.7% of MSM from a 2014 national survey report consistent condom use [72], whereas in our study this was 50.6%. In addition, the national survey found 26.9% of MSM were using psychotropic drugs prior to having sex [72], which is higher than the 17.2% found in our study. The proportion of MSM diagnosed with an STI was higher in our study at 14.2%, compared to the national average of 8.1% [72]. However it is important to note that deviations from national data may have arisen due to our sampling strategy. Our study was restricted to MSM who were HIV negative or unaware of their status, who are the primary candidates for PrEP. While not representative of all MSM in Malaysia, our sample is nevertheless important given the large sample size and their higher risk profiles, which can inform implementation of PrEP and other HIV prevention interventions.

The online methodology used in our study relied on participants’ accurate self-report and honest responses to screening and all survey items, which may have introduced bias. Post-survey measures to verify the self-report information were not employed, and would have had additional ethical implications. For instance, some participants may have misrepresented themselves as Malaysian citizens. Nevertheless, these findings will be useful for HIV prevention among MSM in Malaysia regardless of citizenship status.

In terms of analysis, the dichotomization of continuous variables adopted in our study could have reduced the power to identify associations, between willingness to use PrEP and dichotomized variables [73]. However, this strategy was only employed in three attitudinal and non-biological measures, i.e. likelihood of contracting HIV, confidence in practising safer sex, and communication with healthcare providers; it was not used for all continuous variables. The question about ‘chemsex’ did not specify the type of recreational drugs in the sexual context and participants may have considered a range of drugs when reflecting on whether they had engaged in this behavior, including the wide range of psychotropic stimulants, nervous system depressants, and erectile dysfunction medications mentioned in the preceding survey question. When preparing sexual health and harm reduction interventions for men who use drugs, it is important to take into account differences in motivation, psychological effect, and impact on sexual behavior risk taking between these drugs [74, 75], which was inadequately accounted for in our study. A more granulated approach to the use of drugs during sex should be used in future research. In our multivariable analysis, we included paying for sex but excluded selling sex to men, in light of the results from bivariate analysis. However, selling sex to men may place male sex workers at heightened risk of HIV [76]. Future research should explore PrEP specifically among this sub-group of MSM, as has been the case in other countries [77]. Besides selling sex, important psychosocial variables such as perceived stigma related to taking HIV PrEP [46], and providers’ attitudes on PrEP and their patients [78] which are important determinants in uptake of PrEP were not considered in our study. In the context of civil law (penal code) and sharia law against homosexuality in Malaysia [58], MSM are criminalized and stigmatized.

The scale to determine willingness to use PrEP was adopted from Holt et al.’s study [27] which used a seven-item scale. Given the fewer scale items in our study, the use of a similar cut off as that used in Holt et al.’s study [27] (of 4) may have resulted in a different proportion of MSM willing to use PrEP in our study. However, the cut off of 4 was considered meaningful, while the scale was shorter. Retrospectively, we found that the grand mean and the grand standard deviation were 3.46 and 1.03 respectively. Out of the five items, 3 had median score of 4. These findings suggest that there was limited variation in the scale and the cut off of 4 was suitable. Nevertheless, reported levels of willingness to use PrEP may be affected by the fact that half of the participants did not know or were unsure of the difference between PrEP and PEP even after detailed descriptions were provided.

## Conclusion

This study demonstrates a relatively low awareness of, and willingness to use, PrEP among MSM in Malaysia. However, MSM who were aware of PrEP and who reported HIV risk-related behaviors were more likely to report a willingness to use PrEP were it to become available. Our findings underscore the need to promote the awareness and understanding of PrEP as an effective and safe prevention tool, in combination with other safer sex methods that are appropriate given an individuals’ personal circumstances. Additionally, national policies and implementation tools are needed to support successful implementation of PrEP. Public health efforts need to focus on how to increase access of basic HIV-related services for MSM and consider how best to integrate PrEP within existing services.

## Supporting information

S1 File PrEP Questionnaire. (DOCX)
