# Supplementary material for: Willingness to use pre-exposure prophylaxis for HIV prevention among men who have sex with men in Malaysia: Findings from an online survey
Source: PLoS One. 2017 Sep 13;12(9):e0182838. doi: 10.1371/journal.pone.0182838 (PMC5597127; doi:10.1371/journal.pone.0182838)
Supplement: S1 File — (DOCX) [file pone.0182838.s001.docx]

**WILLINGNESS TO USE PrEP QUESTIONNAIRE**

You are invited to participate in a survey about safer sex **among men who have sex with men (MSM)**. There is now a new possible way of preventing yourself from getting HIV. This is called **Pre-Exposure Prophylaxis (PrEP)**

In this survey, we would like to explain to you about how PrEP works and then ask you what you think about it. This will help us understand what the challenges might be in making PrEP available for Malaysian MSM in the future.

In this current research study, we are only trying to collect information from people who believe themselves to be **HIV negative**.

***If you are HIV positive, thank you for your interest but we cannot collect information from you at this time.***

This is a research project being conducted by the Centre of Excellence in AIDS University Malaya, the Malaysian ADIS Council and the International HIV/ AIDS Alliance.

The survey should take you approximately **20 to 30 minutes** to complete. Your participation is voluntary and you can exit the survey at any time.

**PARTICIPATION**
Your participation in this survey is voluntary. You may refuse to take part in the research or exit the survey at any time without penalty.

**BENEFITS**
You will receive no direct benefits from participating in this online research study.

**RISKS** 
There are no foreseeable risks involved in participating in this study

**CONFIDENTIALITY**
Your survey answers will be sent to a link at SurveyMonkey.com where data will be stored in a password protected electronic format. Survey Monkey does not collect identifying information such as your name, email address, or IP address. Therefore, your responses will remain anonymous. No one will be able to identify you or your answers, and no one will know whether or not you participated in the study. 

At the end of the survey you will be asked if you are interested in participating in a focus group. If you choose to provide contact information such as your phone number or email address, your survey responses may no longer be anonymous to the researcher. However, no names or identifying information would be included in any publications or presentations based on these data, and your responses to this survey will remain confidential.

**CONTACT**
If you have questions at any time about the study or the procedures, you may contact the Principle Investigator, Dr. Iskandar via email at iskandar.azwa@gmail.com

If you feel you have not been treated according to the descriptions in this form, or that your rights as a participant in research have not been honoured during the course of this project, or you have any questions, concerns, or complaints that you wish to address to someone other than the investigator, you may contact Dr.Howie Lim at [Howie.ceria@gmail.com](mailto:Howie.ceria@gmail.com) or Ms.Joseyln Pang atjoselyn@mac.org.my

By clicking on the “Agree” button indicates that 

- You have read the above information 
- You voluntarily agree to participate

- You have never taken this survey before
- You are a Malaysian who is 18 years of age or older

- You identify yourself as Men who has sex with Men (MSM)

-You currently live in Malaysia
- You do not have HIV (HIV negative) or unsure of your HIV status

( ) Agree 

( ) Disagree

**DEMOGRAPHICS**

1. **What is your nationality?**
2. Malaysian national
3. Permanent Residents (exit Survey)
4. Foreigner (exit Survey)
5. Year of birth:
6. **Where are you currently residing?**
7. Kuala Lumpur
8. Labuan
9. Putrajaya
10. Johor
11. Kedah
12. Kelantan
13. Melaka
14. Negeri Sembilan
15. Pahang
16. Perak
17. Perlis
18. Penang
19. Sabah
20. Sarawak
21. Selangor
22. Terengganu
23. **How do you describe your race / ethnicity?**
24. Malay
25. Chinese
26. Indian
27. Orang Asli of Sabah
28. Orang Asli of Sarawak
29. Other (please specify) __________
30. **What is your gender Identity?**
31. Male
32. Transgender Female (exit survey)
33. Transgender Male (exit Survey)
34. Other (please specify) (exit survey)
35. **What is your sexual identity?**
36. PLU / Homosexual / Gay
37. Bisexual
38. Straight / Heterosexual
39. Don’t know
40. Other (please specify) __________
41. **What is your relationship status?**
42. Single
43. Married to a female
44. In a relationship with a male
45. In a relationship with a female
46. In a relationship with a transgender
47. Divorced
48. Widowed
49. Other (please specify) __________
50. **What is your highest level of education?**
51. Primary
52. Secondary/SPM
53. Undergraduate/University
54. Postgraduate
55. Other (please specify)
56. **What is your employment status?**
57. Student
58. Full Time
59. Part Time
60. Self Employed
61. Unemployed
62. Retired
63. Other (please specify)
64. **What is your monthly income?**
65. No stable income
66. Less than RM2000
67. RM2001 – RM3000
68. RM3001 – RM4000
69. RM4001 – RM5000
70. RM5001 – RM6000
71. RM6001 – RM7000
72. RM7001 – RM8000
73. RM8001 – RM9000
74. RM9001 – RM10,000
75. More than RM10,001

**QUESTIONS ABOUT STI TESTING**

1. **Have you ever taken an HIV test?**
2. Yes
3. No (Skip to Q16)
4. **When was you last test?** (Month / Year)
5. **Where did you get tested?**
   1. Government Clinic
   2. Government Hospital
   3. Private clinic
   4. Private hospital
   5. Non-Government organisation
   6. Self test
6. **What was the result of your most recent HIV test?**
7. Negative
8. Positive (Exit Survey)
9. Don’t know/Unsure
10. **How often do you test for HIV?**
    1. Every 3 months
    2. Every 6 months
    3. Once a year
    4. Every two years
    5. I have never tested for HIV
    6. Others (please specify) __________
11. **To what extent do you agree with the following statements?**

16.1 “It is likely I will contract HIV within the next 12 Months”

- - 1. Strongly Agree
    2. Agree
    3. Not Sure
    4. Disagree
    5. Strongly disagree

16.2 “The sex I have is always as safe as I want it to be.”

**For the purpose of this study, we define sex as Anal and/or Virginal Sex only**

1. Strongly Agree
2. Agree
3. Not Sure
4. Disagree
5. Strongly disagree

16.3 “I feel comfortable telling my doctor about my sexual behaviour”

1. Strongly Agree
2. Agree
3. Not Sure
4. Disagree
5. Strongly disagree
6. **In the last 12 months have you been diagnosed with a sexually transmitted infection (STI) other than HIV?**
7. Yes
8. No
9. I don’t know
10. What STI were you positive for? (Click all that applies)
    1. Syphilis
    2. Chlamydia
    3. Gonorrhoea
    4. Hepatitis B
    5. Hepatitis C
    6. Herpes
    7. Human Papillomavirus (HPV)/Genital Warts
    8. Others (Please specify)
    9. Not Sure

**Sex**

**Sex with Male**

1. **In the last 6 months, have you had anal sex with male/s?**
2. Yes
3. No
4. **In the last 6 months I have:**
5. Been Fucked (bottom)
6. Fucked Only (top)
7. Fuck and been fucked (versatile)
8. None of the above
9. **In the past 6months, have you had only ONE male sexual partner?**
10. Yes
11. No
12. **Do you have the same HIV status as your male sexual partner?**
13. Yes, we are both HIV positive (exit survey)
14. Yes, we are both negative
15. He is HIV Negative and I am HIV Positive (Exit survey)
16. He is HIV Positive and I am HIV Negative
17. I don’t know
18. **How often do you use condoms with this male sexual partner?**
19. Always
20. Often
21. Sometimes
22. Rarely
23. Never
24. **How often do you use water/silicon based lubricants for anal sex with this male sexual partner?**
25. No anal sex
26. Always
27. Often
28. Sometimes
29. Rarely
30. Never
31. **How many other sexual male partners have you had sex with in the previous 6 months other than with this male sexual partner?**
32. None
33. 1-3
34. 4-10
35. 11-20
36. More than 21
37. **How many sexual male partners have you had anal sex with in the previous 6 months?**
38. 2-3
39. 4-10
40. 11-20
41. More than 21
42. **Do you have the same HIV status as your other sexual partners?**
43. Yes, we are all HIV positive (exit survey)
44. Some (one or more) are HIV Positive
45. Yes, we are all negative
46. They are all HIV Negative and I am HIV Positive (Exit survey)
47. They are all HIV Positive and I am HIV Negative
48. I don’t know
49. **How often do you use condoms with these male sexual partners?**
50. Always
51. Often
52. Sometimes
53. Rarely
54. Never
55. **How often do you use water/silicon based lubricants for anal sex with your male sexual partners?**
56. No anal sex
57. Always
58. Often
59. Sometimes
60. Rarely
61. Never

**Sex with Female**

1. **In the last 6 months, have you had vaginal and/or anal sex with female/s?**
2. Yes
3. No
4. **In the last 6 months I have had the following type of sex with my female sexual partner:**
5. Vaginal sex
6. Anal Sex
7. Both Vaginal and Anal sex
8. **In the past 6 months, have you had only one female sexual partner?**
9. Yes
10. No
11. **Do you have the same HIV status as your female sexual partner?**
12. Yes, we are both HIV positive (Exit survey)
13. Yes, we are both negative
14. She is HIV Negative and I am HIV Positive (Exit Survey)
15. She is HIV Positive and I am HIV Negative
16. I don’t know
17. **How often do you use condoms with your female sexual partner during vaginal and/or anal sex?**
18. No Vaginal and / or Anal Sex
19. Always
20. Often
21. Sometimes
22. Rarely
23. Never
24. **How many other female sexual partners have you had vaginal and/or anal sex with in the previous 6 months besides this female sexual partner?**
25. None
26. 1-3
27. 4-10
28. 11-20
29. More than 21
30. **How many other female sexual partners have you had vaginal and/or anal sex with in the previous 6 months?**
31. 2-3
32. 4-10
33. 11-20
34. More than 21
35. **Do you have the same HIV status as your other Female sexual partners?**
36. Yes, we are all HIV positive (exit survey)
37. Some (one or more) are HIV Positive
38. Yes, we are all negative
39. They are all HIV Negative and I am HIV Positive (Exit survey)
40. They are all HIV Positive and I am HIV Negative
41. I don’t know
42. **How often do you use condoms with your female sexual partners during vaginal and/or Anal sex?**
43. No Vaginal and/or Sex
44. Always
45. Often
46. Sometimes
47. Rarely
48. Never

**Sex with Transgender Female**

1. **In the last 6 months, have you had sex with Female Transgender/s?**

**(For the purpose of this study, we define sex as Anal and/or Virginal Sex only)**

1. Yes
2. No
3. **In the last 12 months I have:**
4. Been Fucked (Bottom)
5. Fucked Only (top)
6. Fuck and been fucked (versatile)
7. None of the above
8. **In the past 12 months, have you had only one female transgender sexual partner?**
9. Yes
10. No
11. **Do you have the same HIV status as your female transgender sexual partner?**
12. Yes, we are both HIV positive (Exit survey)
13. Yes, we are both negative
14. She is HIV Negative and I am HIV Positive (Exit Survey)
15. She is HIV Positive and I am HIV Negative
16. I don’t know
17. **How often do you use condoms with your female transgender sexual partner during vaginal and/or Anal sex?**
18. No Vaginal and/or Anal Sex
19. Always
20. Often
21. Sometimes
22. Rarely
23. Never
24. **How many other female sexual partners have you had vaginal and/or anal sex with in the previous 6 months besides this female transgender sexual partner?**
25. None
26. 1-3
27. 4-10
28. 11-20
29. More than 21
30. **How many other female transgender sexual partners have you had vaginal and/or anal sex with in the previous 6 months?**
31. 2-3
32. 4-10
33. 11-20
34. More than 21
35. **Do you have the same HIV status as your other Female transgender sexual partners?**
36. Yes, we are all HIV positive (exit survey)
37. Some (one or more) are HIV Positive
38. Yes, we are all negative
39. They are all HIV Negative and I am HIV Positive (Exit survey)
40. They are all HIV Positive and I am HIV Negative
41. I don’t know
42. **How often do you use condoms with your female transgender sexual partner during vaginal and/or Anal sex?**
43. No Vaginal and/or Anal Sex
44. Always
45. Often
46. Sometimes
47. Rarely
48. Never

**QUESTIONS ABOUT DRUG USE**

1. Have you ever taken any party / recreational / illicit drug? (e.g. Cannabis (marijuana, weed), Cocaine Foxy (“F), GHB/GBL (“G”) ,Heroin, “Ice”, LSD, Magic mushroom, Poppers, Viagra/Cialis)
2. Yes
3. No

60.1: What drugs do you use? Click all that applies

a) Cannabis (marijuana, weed),

b) Cocaine

c) Foxy (“F),

d) GHB/GBL (“G”) ,

e) Heroin, “

f) Ice”,

g) LSD,

h) Magic mushroom,

i) Poppers,

j) Viagra/Cialis

k) Others (Please state)

1. In the past 6 months, how often have you taken recreational drugs before or during sex (chem sex)?**(We define sex as Anal and/or Virginal Sex only)**
2. Never
3. Less than a month
4. Once a week
5. 2-3 times a week
6. Daily
7. In the past 6 months, how did you take the recreational drugs?
8. Oral/swallowing/drinking
9. Smoking/using bong
10. Nasal/snorting
11. Anal/booty bumping
12. Injecting
13. In the last 6 months, how many percent(%) of the sex you’ve had was after taking party / recreational / illicit drug?**(For the purpose of this study, we define sex as Anal and/or Virginal Sex only)**
14. Rating: 0% to 100%

**QUESTIONS ABOUT PrEP**

1. **Have you ever heard of Pre-Exposure Prophylaxis (PrEP)?**
2. Yes
3. No
4. Not sure I don’t know (Skip to statement about PrEP)
5. **What is PrEP? (click all that applies)**
6. It’s a pill you take before unsafe sex (No condom used)
7. It’s a pill taken after unsafe sex (No condom used)
8. It’s a pill you take before safer sex (Condom used)
9. It’s a pill taken after safer sex (Condom used)
10. It’s a pill you take everyday
11. It’s a pill you take so that you can have sex without condom
12. It’s a pill you take that prevents other STI other than HIV.
13. **Where did you first learn about PrEP?**
14. Newspaper and magazines
15. Internet
16. Professional journals
17. From friends
18. Doctors
19. Other (please specify) __________
20. **Have you ever used PrEP?**
21. Yes
22. No
23. **Where did/do you get your PrEP from?**
24. Local Private GP
25. Local Private Hospitals
26. Infectious Disease Clinic
27. Community based clinic/Klinik Kesihatan
28. Thai Red Cross Clinic, Bangkok
29. Others (please specify)
30. **Why did you choose PrEP as an HIV prevention option? (Tick all that apply)**
31. I am in a sero-discordant relationship (my sexual partner is HIV positive)
32. My sexual partner/s choose/s not to use condoms consistently
33. I do not like to use condoms
34. I do not always have access to condoms
35. To reduce anxiety about becoming HIV positive
36. I consider myself at high risk of HIV infection
37. Other (please specify) __________
38. **Whilst on PrEP, did/do you test regularly (every 3 or 6 months) for the following? (Tick all that apply)**
39. HIV
40. Hepatitis B
41. Kidney Function
42. Sexual Transmitted Infections (STI / STD)
43. Other (please specify)
44. No regular testing
45. **Whilst on PrEP, how often did/do you use condoms?**
46. Always
47. Often
48. Sometimes
49. Rarely
50. Never
51. **My experience of using PrEP is / was**
52. Very good
53. Satisfactory
54. Neutral
55. Unsatisfactory
56. Very Bad
57. You had answered your experience with PrEP is/was unsatisfactory or very bad in the last question. Why is that? (Click all the applies)
    1. Bad Side effects
    2. Difficult to get access to PrEP
    3. The cost of PrEP was high
    4. Others (Please specify)
58. **Are you currently still taking PrEP daily**
59. Yes
60. No
61. You had answered you no longer take PrEP daily. Why is that? (Click all the applies)
    1. Bad Side effects
    2. Difficult to get access to PrEP
    3. The cost of PrEP was high
    4. I take a lower weekly dose
    5. I always use a condom
    6. I’m worried PrEP won’t work and I will get HIV
    7. I’m worried about forgetting to take my medication
    8. I’m worried about what other people might think of me
    9. I am currently not having regular sex
    10. Others (Please specify)
62. **Is the following statement true or false:**

**PrEP does not protect you against other STIs and blood borne viruses such as Syphilis, Gonorrhoea, Hepatitis A, B and C?**

1. True
2. False
3. Not Sure

**------------------------------------------------------------------------------------------------------------------------**

**We want to ensure that everyone who completes this survey has the same level of information when they do this survey. To help ensure that, here is a short summary about PrEP that explains some important details.**

**"*PrEP is a daily medication that people who do not have HIV take to prevent getting infected with HIV. PrEP is taken before someone is exposed to HIV. While PrEP is not yet available in Malaysia, it is thought that PrEP will probably be of most benefit to people who perceive themselves to be at a higher risk of contracting HIV at certain points in their lives. PrEP could have more optimal benefit if it is used together with other methods of preventing HIV. However, it may also be useful for people who have experienced difficulty in using condoms consistently. PrEP works best if you take it every day and while there can be some side-effects at first (such as nausea and headaches) these generally reduce after a few weeks of use. People who take PrEP should have regular sexual health check-ups, including HIV testing to ensure the medication is working*."**

1. **Thinking about you just read about pre−exposure prophylaxis (PrEP), how much do you agree or disagree with the following statement?**
   1. “I would need to take PrEP.”
      1. Strongly disagree
      2. Disagree
      3. Neither agree nor disagree
      4. Agree
      5. Strongly agree

77.2 “I would take PrEP even if it wasn't 100% effective.”

a) Strongly disagree

b) Disagree

c) Neither agree nor disagree

d) Agree

e) Strongly agree

77.3 “I am going to take PrEP as soon as it becomes available.”

a) Strongly disagree

b) Disagree

c) Neither agree nor disagree

d) Agree

e) Strongly agree

- 1. “I would take pills before and after sex if it would prevent me getting HIV.”
     1. Strongly disagree
     2. Disagree
     3. Neither agree nor disagree
     4. Agree
     5. Strongly agree

77.5 “I would take a pill every day if it would prevent me getting HIV.”

1. Strongly disagree
2. Disagree
3. Neither agree nor disagree
4. Agree
5. Strongly agree

77.6“If I took PrEP I would be willing to test for HIV at least four times a year.”

1. Strongly disagree
2. Disagree
3. Neither agree nor disagree
4. Agree
5. Strongly agree

77.7“I would be willing to pay for PrEP.”

1. Yes
2. No
3. Don’t know/not sure
4. **How much would you be willing to spend on PrEP per month?**
5. Less than RM100
6. Between RM101 and RM200
7. BetweenRM201 and RM300
8. Between RM301 and RM400
9. Between RM401 and RM500
10. More than RM500
11. **If you are unwilling or unable to pay for PrEP as an HIV prevention option, which institutions would you expect to cover the cost? (Tick all that apply)**
12. Government
13. Private Health Insurance
14. Other (please specify) __________
15. **If PrEP was made available in Malaysia, how likely do you think you are to use it??**
16. Very likely
17. Quite likely
18. Not sure
19. Not very likely
20. Very unlikely
21. **Why do you think you are likely to use PrEP as an HIV prevention option?(Tick all that apply)**
22. To prevent catching HIV
23. I have problems wearing condoms
24. To take responsibility for my sexual health
25. I have problems getting my partner to agree to use condoms
26. To feel more in control of my sexual health
27. To protect my family
28. I feel shy purchasing condoms
29. Other reason (please specify) ­­­­__________
30. **If PrEP was to be made available as a HIV prevention option, where would you prefer to access it?**
31. Private GP
32. Private Hospital
33. Government Clinic / Hospital (Infectious Disease Clinic/ART treatment centres)
34. Community Based health Clinics/Klinik Kesihatan
35. Community based organisation (e.g. PT Foundation / KLASS/ Intan Life Zone/ Fhada)
36. Somewhere else(please specify) __________

(Skip to statement about PEP)

1. **Why do you think it is Unsure /unlikely you would use PrEP as an HIV prevention option? (Tick all that apply)**
2. I’m worried about possible side effects
3. I always use a condom
4. I’m worried PrEP won’t work and I will get HIV
5. I’m worried about forgetting to take my medication
6. I’m worried about what other people might think of me
7. I can’t afford PrEP
8. Other (please specify) __________
9. A recent study found that taking 2 PrEP pills (Truvada)between 2 and 24 hours prior to having sex with another man, followed by another two pills 24 and 48 hours after sex was highly effective in preventing HIV infection.

Some people may choose to take PrEP only when they think they will have high risk behaviour for HIV infection, rather than each day.

Which statement best describes your opinion?

1. I will continue taking PrEP every day
2. I will only take PrEP before and after high-risk sex
3. I am unsure about when and how to take PrEP

**QUESTIONS ABOUT PEP**

1. Have you heard about PEP (Post-Exposure Prophylaxis)?
2. Yes
3. No

**We want to ensure that everyone who completes this survey has the same level of information when they do this survey. To help ensure that, here is a short summary about PEP that explains some important details.**

**“Post Exposure Prophylaxis (PEP) is the use of antiretroviral drugs after a single high-risk event to stop HIV from making copies of itself and spreading through your body. PEP must be started as soon as possible to be effective—and always within 3 days of a possible exposure. “**

1. Have you taken PEP within the past 12 months?
2. Yes
3. No
4. Where did you get your PEP from?
5. Private GP/ Hospitals
6. Government Clinic (infectious Disease Clinic)
7. Community Based health Clinic/Klinik Kesihatan
8. Other (please specify) __________
9. Do you understand the difference between PrEP and PEP?
10. Yes
11. No
12. Unsure

**To recap, this is what PrEP and Pep Is**

**"*PrEP is a daily medication that people who do not have HIV take to prevent getting infected with HIV. PrEP is taken before someone is exposed to HIV. While PrEP is not yet available in Malaysia, it is thought that PrEP will probably be of most benefit to people who perceive themselves to be at a higher risk of contracting HIV at certain points in their lives. PrEP could have more optimal benefit if it is used together with other methods of preventing HIV. However, it may also be useful for people who have experienced difficulty in using condoms consistently. PrEP works best if you take it every day and while there can be some side-effects at first (such as nausea and headaches) these generally reduce after a few weeks of use. People who take PrEP should have regular sexual health check-ups, including HIV testing to ensure the medication is working*."**

**“Post Exposure Prophylaxis (PEP) is the use of antiretroviral drugs after a single high-risk event to stop HIV from making copies of itself and spreading through your body. PEP must be started as soon as possible to be effective—and always within 3 days of a possible exposure. “**

**QUESTIONS ABOUT SETTINGS THAT MSM VISIT**

1. **When did you last visit a gay cruising park gay for anal sex with another Male?**
2. Never
3. Within the last 24 hours
4. Within the last 7 days
5. Within the last 4 weeks
6. Within the last 6 months
7. Within the last 12 months
8. Within the last 5 years
9. More than 5 years ago
10. **When did you last cruise in a public toilet for anal sex with another male?**
11. Never
12. Within the last 24 hours
13. Within the last 7 days
14. Within the last 4 weeks
15. Within the last 6 months
16. Within the last 12 months
17. Within the last 5 years
18. More than 5 years ago
19. **When did you last visit a gay sauna for anal sex with another male?**
20. Never
21. Within the last 24 hours
22. Within the last 7 days
23. Within the last 4 weeks
24. Within the last 6 months
25. Within the last 12 months
26. Within the last 5 years
27. More than 5 years ago
28. **When did you last visit a gay bar / disco for the purpose of finding a male partner for anal sex?**
29. Never
30. Within the last 24 hours
31. Within the last 7 days
32. Within the last 4 weeks
33. Within the last 6 months
34. Within the last 12 months
35. Within the last 5 years
36. More than 5 years ago
37. **When did you last visit a gay sex party in a private home to have anal sex?**
38. Never
39. Within the last 24 hours
40. Within the last 7 days
41. Within the last 4 weeks
42. Within the last 6 months
43. Within the last 12 months
44. Within the last 5 years
45. More than 5 years ago
46. **When did you last visit a gay website / social app to find male/s you could have anal sex with?**
47. Never (Skip to Q96)
48. Within the last 24 hours
49. Within the last 7 days
50. Within the last 4 weeks
51. Within the last 6 months
52. Within the last 12 months
53. Within the last 5 years
54. More than 5 years ago
55. What are the gay websites / social apps you go to for this purpose?
    1. Grindr
    2. Jack’d
    3. Hornet
    4. Planet Romeo
    5. Growlr
    6. Scruff
    7. Facebook
    8. Wechat
    9. Line
    10. Telegram
    11. Others (Please specify)
56. **When did you last visit a massage centre to find a masseur you could haveanal sex with?**
57. Never
58. Within the last 24 hours
59. Within the last 7 days
60. Within the last 4 weeks
61. Within the last 6 months
62. Within the last 12 months
63. Within the last 5 years
64. More than 5 years ago

**QUESTIONS ABOUT COMMERCIAL SEX**

1. **Have you ever received money, goods or services in exchange for anal sex with a male partner?**
2. Yes
3. No

1. **Have you ever given money, goods or services in exchange for anal sex with a male partner?**
2. Yes
3. No
4. **Have you ever received money, goods or services in exchange for vaginal and / or anal sex with a female partner?**
5. Yes
6. No

1. **Have you ever given money, goods or services in exchange for vaginal and / or anal sex with a female partner?**
2. Yes
3. No

**Have you ever received money, goods or services in exchange for vaginal and / or anal sex with a female transgender partner?**

1. **Yes**
2. **No**

**Have you ever given money, goods or services in exchange for vaginal and / or anal sex with a female transgender partner?**

1. **Yes**
2. **No**

**QUESTION ABOUT HAPPINESS WITH SEX**

1. **How happy are you with your sex life right now?**
2. **Very happy**
3. **Quite** happy
4. Not sure/don’t know
5. Quite unhappy
6. Very unhappy
7. Would you be interested in participating in a focus group discussion to be held in Kuala Lumpur in March2016?
   1. Yes
   2. No (Go to Q106)
8. Please leave us your contact details as below

Name:

Contact Number:

Email address:

1. Thank you for participating in the survey. If you would like to know where you can get screened for HIV or to do a STI test, do contact 012-9438450 (Whatsapp/Wechat/Line) between Monday – Friday from 9am to 5pm for more information. Thank you.

Disqualify Page

Thank you for your interest in the study but unfortunately you are not eligible to take part this time as you do not fulfil at least one of the following criteria.

- You have read the above information 
- You voluntarily agree to participate

- You have never taken this survey before
- You are a Malaysian who is 18 years of age or older

- You identify yourself as Men who has sex with Men (MSM)

-You currently live in Malaysia
- You do not have HIV (HIV negative) or unsure of your HIV status

If you would like to know where you can get screened for HIV or to do a STI test, do contact 012-9438450 (Whatsapp/Wechat/Line) between Monday – Friday from 9am to 5pm for more information.

Thank you once again for your interest in taking part in the survey.
